# Evaluating the process of practice enhancement for exclusive breastfeeding (PEEB): a participatory action research approach for clinical innovation

**DOI:** 10.1186/s13006-024-00648-7

**Published:** 2024-05-31

**Authors:** Elaine Lehane, Catherine Buckley, Helen Mulcahy, Elizabeth McCarthy, Liz Cogan, Rhona O’Connell, Margaret Murphy, Patricia Leahy-Warren

**Affiliations:** 1 School of Nursing and Midwifery, University College Cork, Cork, Ireland; 2 Northridge House Education and Research Centre, St Luke’s Home, Cork, Ireland

**Keywords:** Exclusive breastfeeding, Participatory action research, Work based learning, Innovation, Breastfeeding

## Abstract

**Background:**

Despite the known benefits of exclusive breastfeeding, global rates remain below recommended targets, with Ireland having one of the lowest rates in the world. This study explores the efficacy of Participatory Action Research (PAR) and Work-Based Learning Groups (WBLGs) to enhance breastfeeding practices within Irish healthcare settings from the perspective of WBLG participants and facilitators.

**Methods:**

Employing a PAR approach, interdisciplinary healthcare professionals across maternity, primary, and community care settings (*n* = 94) participated in monthly WBLGs facilitated by three research and practice experts. These sessions, conducted over nine months (November 2021 – July 2022), focused on critical reflective and experiential learning to identify and understand existing breastfeeding culture and practices. Data were collected through participant feedback, facilitator notes, and reflective exercises, with analysis centered on participant engagement and the effectiveness of WBLGs. This approach facilitated a comprehensive understanding of breastfeeding support challenges and opportunities, leading to the development of actionable themes and strategies for practice improvement.

**Results:**

Data analysis from WBLG participants led to the identification of five key themes: *Empowerment, Ethos, Journey, Vision*, and *Personal Experience.* These themes shaped the participants’ meta-narrative, emphasising a journey of knowledge-building and empowerment for breastfeeding women and supporting staff, underlining the importance of teamwork and multidisciplinary approaches. The project team’s evaluation highlighted four additional themes: *Building Momentum, Balancing, Space Matters*, and *Being Present.* These themes reflect the dynamics of the PAR process, highlighting the significance of creating a conducive environment for discussion, ensuring diverse engagement, and maintaining energy and focus to foster meaningful practice changes in breastfeeding support.

**Conclusion:**

This study highlights the potential of WBLGs and PAR to enhance the understanding and approach of healthcare professionals towards breastfeeding support. By fostering reflective and collaborative learning environments, the study has contributed to a deeper understanding of the challenges in breastfeeding support and identified key areas for improvement. The methodologies and themes identified hold promise to inform future practice and policy development in maternal and child health.

## Background

Breastfeeding results in positive health and well-being outcomes for maternal and infant health, population health in reducing risk of chronic diseases; environmental sustainability, food hygiene, economic savings, workplace productivity and reducing health disparities. Despite this evidence, internationally breastfeeding rates vary significantly, with rates in Ireland being the lowest in Europe. Breastfeeding results in positive health and well-being outcomes for society [1, 2]. Exclusive breastfeeding (EBF) is defined as giving breast milk only to the infant, without any additional food or drink, not even water in the first six months of life, apart from mineral supplements, vitamins, or medicines [3]. The World Health Organization (WHO) recommends that infants are exclusively breastfed for the first six months with continued breastfeeding up to 2 years and beyond [4]. The global exclusive breastfeeding target is to increase the rate of exclusive breastfeeding in the first 6 months up to at least 50% by 2025 [5] and 70% by 2030 [6]. Global rates of exclusive breastfeeding vary based on population, definitions and the manner in which data is collected. Ireland is recognised as having one of the lowest breastfeeding rates in the world, with just 63% of babies receiving breast milk at birth and < 5% exclusively breastfeeding at 6 months [7]. Whilst there is no nationally recognised dataset in Ireland to monitor exclusive breastfeeding rates beyond three months postpartum, there is recognition that efforts in Ireland to support continued breastfeeding must be amplified significantly to reach the 2030 targets [8].

‘Exclusive’, and ‘any’ breastfeeding duration increases when additional breastfeeding support is provided by health professionals (HPs) and/or peer supporters, antenatally/postnatally and across hospital and community settings [9]. Characteristics of such support include: (1) guidance that is offered universally during the antenatal and postnatal period by a trained professional or lay person; (2) ongoing continuous visits to allow for predictability of care; and (3) support that is tailored to the needs of the population [10]. However, significant challenges exist with providing such support including; misinformation on breastfeeding physiology; lack of evidence-based knowledge and skills from HPs [10]; and inadequate, sustained societal support for exclusive breastfeeding [11].

Support from trained HPs, in conjunction with wider cultural changes within healthcare settings, is essential to improve breastfeeding rates [10, 12]. While evidence-based education across the perinatal journey is required, breastfeeding education tends to be recommended for midwives and public health nurses generally [13] and is specifically recommended in Irish Public Health Nursing (PHN) and midwifery curricular documents [14, 15]. Furthermore, the standardisation of such education internationally is variable and there is less focus on skills-based training [10]. To translate into multi-disciplinary clinical practice, all staff across hospital and community settings require foundational working knowledge of evidence-based breastfeeding best practice to support parents [16]. This will reduce the likelihood of parents receiving incorrect, inaccurate, or inconsistent advice [12]. Pregnant women report striking differences between health promotion messages antenatally, and support received postnatally highlighting a disconnect between implementation of theory into practice [17].

Facilitating the implementation of evidence into practice has been a subject of interest for many years. The publication of policies or research findings does not necessarily guarantee adherence to their recommendations in practice [18–20]. Education alone, although enhancing clinicians’ knowledge and attitudes, is less impactful than practice-based initiatives on patient outcomes [20–22]. There have been calls for the research community to shift the focus from small scale tightly controlled interventions to evaluating those capable of dissemination and translation [23]. Pragmatic trials [24], hybrid effectiveness-implementation trials [25] and participatory research approaches [26] are examples of study designs more suited to community and clinical-relevant research. These approaches engage key stakeholders, prioritise implementation outcomes, and assess the generalisability of intervention effects.

Participatory action research (PAR) sits well with clinical practice development and innovation, as many of its processes can be used to explain practice initiatives and implementation of change [27]. Fundamentally, PAR is a collective, self-reflective inquiry that researchers and participants undertake to understand and improve on the practices in which they participate and the situations in which they find themselves [28]. To date only a small number of studies have used a PAR approach to advance breastfeeding initiation, for women with gestational diabetes [29], women with disabilities [30] and interventions to improve breastfeeding rates [31, 32].

The PAR approach reported in this paper is part of a larger study, the overarching aim of which is to enhance the implementation of evidence-based practice for Exclusive Breastfeeding (PEEB) throughout a pregnant woman’s journey to 3 months postpartum. To facilitate knowledge translation, an onsite PAR project across hospital, primary care and community settings was conducted and involved implementing three key elements, namely: (1) Education and skills based training for staff encountering women across their journey from hospital to community; (2) Facilitating changes in practice aligned to implementing policy supporting exclusive breastfeeding; and (3) Enhancing the environment by creating dedicated space for exclusive breastfeeding mothers amenable to formal support and peer influence.

The aim of this paper is to report on the efficacy of PAR and Work-Based Learning Groups (WBLGs) to enhance breastfeeding practices within Irish healthcare settings from the perspective of WBLG participants and facilitators.

## Methods

### Design and procedure

A PAR approach [33, 34] was applied, where participants and researchers worked together using a systematic process to observe, consider, and act upon issues identified [35, 36]. PAR emphasises collaboration, reflection, and iterative learning. It involves stakeholders as active participants in the research process, not merely as ‘subjects’ but as co-researchers. It is an approach that is responsive and committed in providing solutions to real world problems [37] and has been particularly valuable in health and social care research in providing crucial insights into the barriers and facilitators of implementing evidence-based practices and expanding understanding of complex situations [38–40]. Workplace learning is a core feature of many action-oriented approaches such as participatory research and includes an emphasis on experiential learning [41, 42] and facilitation of critical reflection in the creation of new professional knowledge [43, 44]. Through discussion, collaboration and reflection, the impact of work-based learning is enhanced both in terms of the professional development of the learner and improved working practices [45].

Nine WBLG sessions took place in a maternity healthcare facility convenient to all participants over a 9-month period (November 2021 – July 2022), with session length ranging from 45 to 65 min approximately. The primary objective of the learning groups was to analyse and understand the existing culture and practices around breastfeeding in diverse healthcare settings. They aimed to pinpoint the challenges and opportunities in breastfeeding experiences and awareness, seeking to identify and enhance understanding of the influencing factors, current breastfeeding support and areas for improvement and innovation in promoting breastfeeding. Each WBLG session was supported by two external facilitators: the ‘content or breastfeeding evidence’ expert and an expert PAR researcher in addition to the project’s clinical research nurse. The three researchers had separate roles in facilitating the PAR group: (1) group facilitator; (2) observer of group dynamics; and (3) breastfeeding evidence expert ‘translator’. During the first WBLG session, the facilitators presented participants with a brief overview of the project and findings from the baseline situational analysis which examined staff demographics, experience, readiness to change, perceived breastfeeding competence and environmental characteristics as described in Mulcahy et al. (in submission) [46]. ‘Icebreaker’ and ‘brainstorming’ activities around participant priority areas followed. As the sessions progressed, facilitation activities such as ‘circle of concern/circle of influence’, ‘in and out’ [47] helped in working through case study development and associated action plans on agreed practice change areas. An outline of these sessions focusing on discussion topics and facilitation processes can be seen in Table 1.

**Table 1:** Work based Learning Group Framework for Participatory Action Research Project (PEEB)

Session Details	Structure/Outline of Reflective WBLG	Processes used
**Session 1** ♣ Getting to know guidance document and aspects of exclusive breast feeding. ♣ Agreeing ways of working.	♣ Overview of the guidance document ♣ The development of (learning initiative for exclusive breastfeeding). ♣ Linking the initiative to current care. ♣ How can this be achieved in practice?	♣ Presentation and facilitated discussion. ♣ Claims, concerns, and issues (CCI). ♣ Creative session with participants asking them to address meta-theme. ♣ Agreeing an engagement contract.
**Session 2** ♣ Critically looking at the current workplace culture	♣ Re-engagement with (learning initiative) and gaining a more in-depth insight. ♣ Working with claims concerns and issues from previous session. ♣ Looking at observations of practice. Identifying how we can work with these observations to improve exclusive breastfeeding	♣ Reflection on issues identified in claims, concerns and issues. ♣ Creating a landscape of the workplace culture. ♣ Identifying strategies to improve exclusive breast feeding.
**Session 3** ♣ Identifying ways to promote exclusive breastfeeding	♣ Recap on (learning initiative) (ask any member of staff for their understanding). ♣ Interviews with mothers & fathers (these will have been done between day 2 and day 3). ♣ Relooking at and reflecting on claims concerns and Issues from day 1.	♣ Staff explain to others their understanding of (learning intervention) and how they would operationalise(use) it practice. ♣ Group work to look at interviews and identify key themes. Developing a shared vision for exclusive breastfeeding ♣ Linking this to the CCI and observations from previous sessions.
**Session 4** ♣ Using data collected to devise action plans.	♣ Work based learning activities how did they go and what were the outcomes. ♣ Looking at strategies from Day 2 and discussion from Day 3 and devising action plans to be worked on over next few sessions.	♣ Reflecting on learning and implications of identified practice. ♣ Development of an action plan. (what is an action plan and how do we use it)
**Session 5.** ♣ Reflecting on where we are now and ongoing forward.	♣ Recap on WBL activities. How have reflections gone? ♣ How did interviews with mothers and fathers go? Have they been completed? ♣ Feedback on Informal ward meeting. Feedback from CNM. ♣ Action planning. Discussion and further work.	♣ Reflecting, making sense of and working with the data collected and looking how this informs action cycles. ♣ Getting a sense of taking ownership of actions. ♣ Strategies to overcome difficulties and maximise benefits of implementation
**Session 6.** ♣ Making the intervention real	♣ Recap on learning intervention ♣ Recap on WBL activities. ♣ Discussion on the data from Documentary analysis. ♣ Action planning. What has been achieved? And how?	♣ Making exclusive breastfeeding real using it in everyday language and continuing to build knowledge of how the (learning intervention) works. ♣ Gain an understanding of how to use data collected. ♣ Looking at what you see happening/the way things are being done in the analysis and how things should be done. ♣ Reflecting on learning and implications for ongoing activities, including the further development of action plans.
**Session 7.** ♣ Spirals and actions.	♣ Recap on activities since last day. ♣ Group reflection on how/if the learning intervention doc has improved practice. ♣ Spirals from action cycles looking at other activities that have arisen (has the introduction of the learning intervention led to other activities being considered) ♣ Looking critically and tweaking documentation (may mean care plans or policies) ♣ Sustainability and going forward	♣ Reflective practice ♣ What is it? ♣ How to get the team involved? ♣ What do I do with mine and other’s reflections? ♣ Facilitated discussion on ways of improving documentation in relation to breastfeeding.
**Session 8** ♣ Evaluation	♣ Reflection on work-based activities that have been taking place since last session. ♣ Evaluation of taking part in the study.	♣ Creative exercise to determine how everyone felt about taking part and to look at changes in practice (if any).
**Session 9** ♣ Celebration and analysis of the WBLGs	♣ Celebration and achievement	♣ Looking at the data collected during the WBLGs and analysing that in a creative way. ♣ Celebration to focus on aspects of practice that have changed/improved since implementation and facilitators to feedback data from the project to the site

The principles of Collaboration, Inclusion and Participation (CIP) [48] were applied to the WBLGs, such that the terms of engagement, content and processes were reviewed continuously by all team members to move the project forward. All participants were encouraged and given time to express their perspectives, experiences, insights, and concerns, with all voices given equal consideration. The expert PAR facilitator provided prompt questions to ensure that the process did not go beyond scope and provided support to participants to help them to direct the session towards possible actions.

### Setting and participants

The study took place in the Southwestern region of the Republic of Ireland and included participants from acute and community healthcare settings, including a general hospital providing maternity services, two General Practitioner (GP) practices, and one Primary Care Facility, which includes public health nurses and allied health professionals. Staff were invited to participate via a recruitment poster in the respective study sites and ‘on the ground’ recruitment by a clinical research nurse, who promoted engagement from the four clinical sites through liaising with senior clinicians and management. In accordance with national (Health Service Executive (HSE)) [49] and international (WHO/UNICEF) recommendations *all* hospital staff should encourage and enable mothers to breastfeed and in doing so be orientated to infant feeding policy and receive training relevant to their role and responsibility. Therefore, any staff member that a woman encountered who could influence her breastfeeding experience were deemed eligible to participate. The WBLGs were attended by interdisciplinary clinical healthcare staff e.g. nursing, midwifery, general practitioners, obstetricians, and service support staff including catering, hygiene services, porters, and administration. For example, clerical staff speak with expectant mothers regularly regarding clinic bookings. By having a knowledge of breastfeeding within the hospital they can signpost women to the various supports available. Similarly, catering staff will recognise the value of frequent healthy snacks for mothers and the impact of missed meals. Patient and Public Involvement (PPI) representatives including two mothers with new-born babies also attended (Table 2). Attendance at WBLGs ranged from 7 to 15 participants (excluding facilitators) with a total of 94 attendees across 9 sessions.

**Table 2:** Work Based Learning Group Profile

	*N* (%)
Gender	
Male Female Total attendees	7 (7) 87 (93) 94 (100)
Setting	
Tertiary Maternity Service Community Primary Care/GP Public & Patient Participation (PPI)	58 (62) 14 (15) 12 (13) 10 (11)
**Profession/Job Title** PPI/Breastfeeding Mother Caterer Clinical Midwife Manager (1 & 2) Clinical Midwife Manager 3 & Assistant Director Midwifery (Practice Development) Clinical Nurse Manager (1 & 2) Business Manager (Maternity Directorate) Service Administrator Community Midwife Community Nurse Registered Midwife (Hospital) Infant Feeding Coordinator Paediatric Registrar Porter Public Health Nurse Student Public Health Nurse Consultant (Obstetrics) Consultant (Paediatrics) General Practitioner (GP) General Practitioner (Trainee) Housekeeping Candidate Advanced Midwife Practitioner Senior House Office (Paediatric /Obstetrics) Registered General Nurse (Mat., Special Care, Gyn) General Practice Nurse Health Care Assistant (maternity) Student Nurse Clinical Skills Facilitator	**(No. of Attendances)** 10 1 6 2 4 2 4 3 4 4 5 1 2 7 2 2 2 7 2 2 1 2 9 2 6 1 2

### Data generation

Data from WBLGs were collected through participant feedback, facilitator field notes, and reflections post-sessions. Eight WBLG session notes captured participant experiences and workplace contexts, focusing on practices supporting breastfeeding. Participants engaged in creative and reflective exercises to discuss and assess their observations and supportive breastfeeding practices. Facilitator field notes included a summary of debriefing, assessing the effectiveness of various facilitation strategies, alongside 16 structured reflections aimed at fostering objectivity in knowledge construction, as outlined by Polit & Beck [50]. These reflections included observations, thoughts, feelings, and personal evaluations by facilitators, focusing on situational elements and group dynamics. This approach was instrumental to gather deeper insights into the action research process, highlighting challenges and enablers of practice change. Finally, a critical part of data collection involved evaluating participant experiences in WBLGs, specifically conducted in the ninth session.

### Data analysis

Part 1 of the analysis focused on assessing participant engagement and the outcomes resulting from their involvement in the research process. Part 2 involved a comprehensive evaluation of the effectiveness of the WBLGs within a PAR framework for enhancing practice. The project team conducted this phase using the same creative analysis methods employed by the WBLG participants, ensuring fidelity and trustworthiness in the analysis process.

**Fig. 1:**
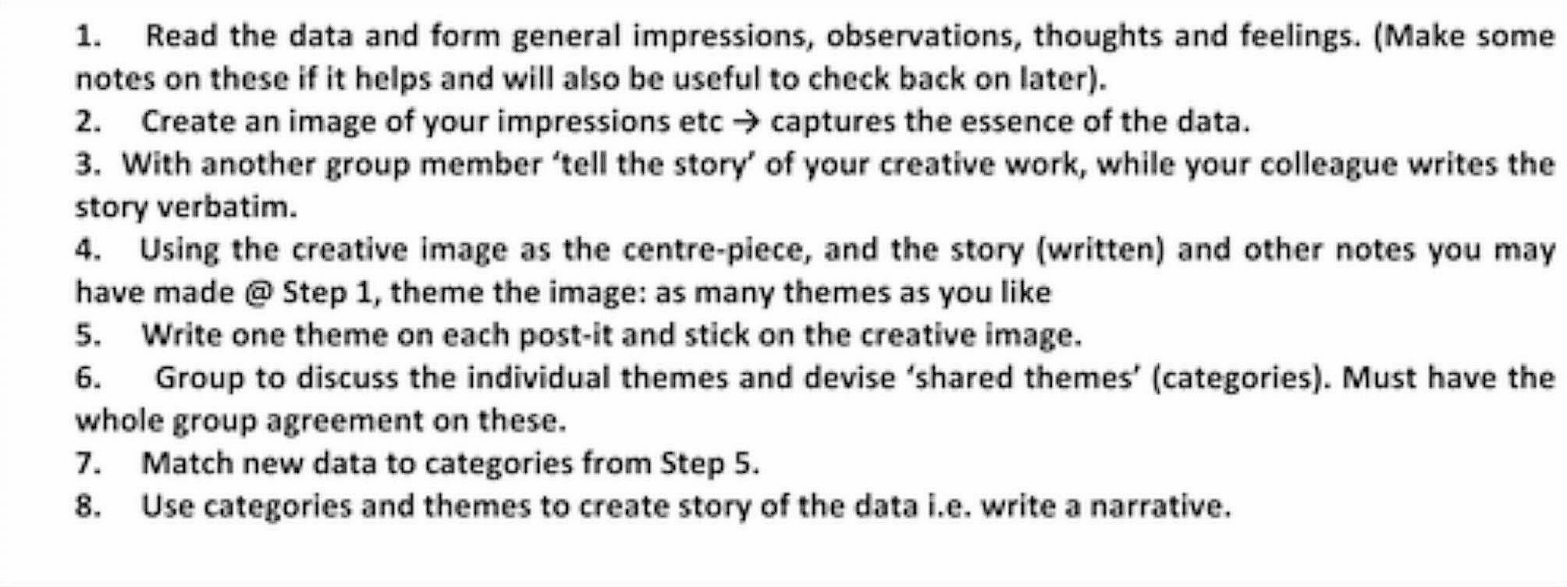
Figure 1 Creative hermeneutic data analysis (Boomer & McCormack, 2010)

A key methodological and ethical issue within participatory action research relates to the analysis and interpretation of the data. Data can be interpreted differently by different partners leading to questions as to who has the “right” interpretation [51]. Considering the power differential that can be present between researchers, facilitators, participants, and other partners, adopting a creative hermeneutic approach fosters an open dialogue to understand the meaning of data across different stakeholders [52]. The use of creative arts can lead to new interpretations and ways of working and in analysis can highlight patterns, themes, and connections [53]. Data analysis from the WBLGs was both ongoing (happening at each WBLG session day with participants) and overarching (at the end of the study with the learning group participants (WBLG 9) and with the facilitators). Using this artistic method; the expert PAR researcher engaged both the participant and facilitator groups in eight stages of individual and group analysis processes, as outlined in Fig. 1. The first steps involved all members of each group looking at the raw data and creating an image or creative expression of the data. They next told the story of their image to one other person in the group who wrote the story verbatim. The tellers and writers switched and repeated the process. They themed their images. The group came together and shared all the themes they had devised. Themes were categorised and a set of group themes were developed. Following these stages, final themes were agreed, representing all the data. The final stage represented the group writing a ‘meta-narrative’ representing all the themes.

## Results

The agreed meta-narrative and themes from the WBLG participants are presented first, followed by the meta-narrative and themes derived from analysis of the process conducted by the project team.

**Fig. 2:**
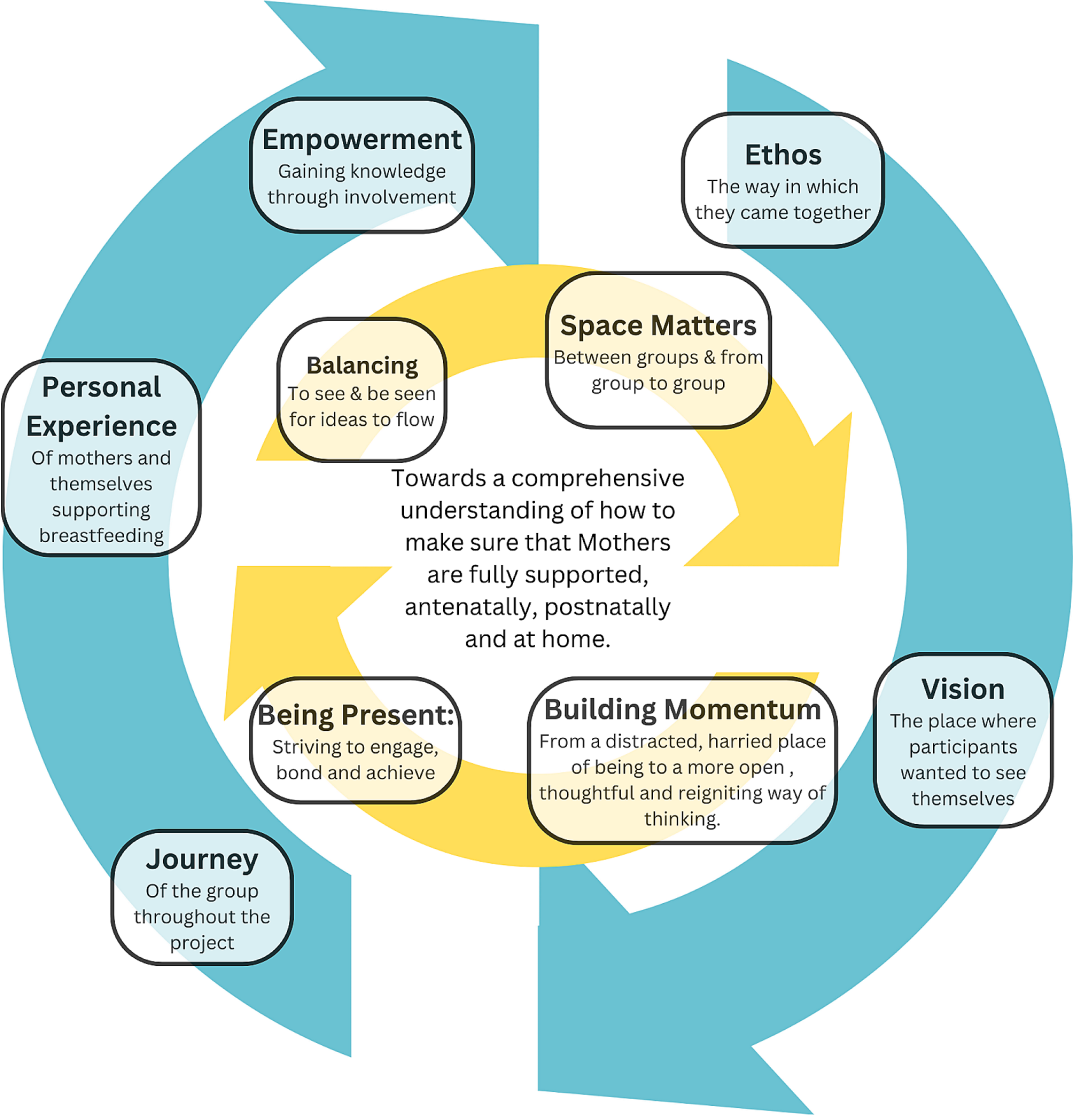
Diagram visualising the process of Practice Enhancement for Exclusive Breastfeeding (PEEB)

Figure 2 is a representation of the themes which emerged from: (1) the WBLG participants and (2) the Project Team, and how they crystallise to the central focus of the study and endorse the view that breastfeeding support is multi-faceted and multidisciplinary. The outer blue dynamic circle contains the themes from WBLG participants; (1) Empowerment, (2) Ethos, (3) Journey, (4) Vision and (5) Personal Experience. The inner yellow dynamic circle captures the project team themes; (1) Building Momentum, (2) Balancing, (3) Space Matters, and (4) Being Present.

### Findings from the WBLG participants

Participants expressed a vision that their role was to empower women on their breastfeeding journey and *‘to promote a calm and peaceful environment’*, which are described under five themes.

***Empowerment*** was identified as gaining knowledge through involvement in the WBLGs. “*Building momentum in small incremental steps”* offered opportunities for participants to reflect on current practice. Participants gained strength from active involvement in the groups through discussing issues and challenging practices that did not support breastfeeding. They also felt the WBLGs offered a safe place to continue gathering strength and building knowledge together with breastfeeding families; “*Everyone was blooming, we were gaining more knowledge”.*

Participants identified a team **Ethos**, that is, uniting as a team to provide mutual support amongst themselves and to breastfeeding mothers as necessary to achieve breastfeeding goals. “*By joining this working group, I learned a lot about the importance of teamwork in helping our breast-feeding moms to get to their own personal goal “; “When we come together as a team – we give a hand”.* It also highlighted the need for *“multifactorial and multidisciplinary approaches”.*

***Journey*** symbolised both the collective path the group followed during the project and the individual experiences of the mothers. Participants stated they “*were on a gradient, moving up, getting and giving support and creating correct environments for breastfeeding”.* There was a sense of “*everyone moving together”* with staff identifying the importance of calmness and environment in changing and improving practice for mothers through the action plans they carried out in their sites. There was support in numbers, with participants having a sense of *“holding hands, sharing experiences, hopes and strengths”*.

***Vision***, articulated early in the project, was defined as the desired end-goal or state that participants aimed to achieve by the project’s conclusion; “*in the end we get a happy Mom and a happy baby”.* Vision was a constant theme and changed and adapted as participants were learning about their own cultures, environments and practices and looking at ways to change these to facilitate a good breastfeeding experience for mothers. The WBLGs enabled participants to “*go from a distracted harried place (hospital/care environment) to a more relaxed thoughtful place and helped focus new ways of thinking/visioning”.* They envisioned places that had *natural beauty*, that were r*elaxed and peacefu*l and offered contentment when breastfeeding, was the *beautiful end.*

At WBLG meetings participants spoke about both the ***personal experience*** of the mothers and of their own personal experiences of supporting breastfeeding in practice. *“When you see mothers coming – the primip (first time mother) - they are lonely and have no idea about breastfeeding – they will always need more help with breastfeeding”.* For some it can be *self-consuming*, and while it is *natural* for some there is the f*ear of the unknown* and of feeling alone. It is a “*rugged, difficult, journey”* for some, with acknowledgement of the need for a *calm and natural approach* by those supporting mothers. For staff they expressed their personal journey in the WBLGs groups and activities as “*taking time to look beyond their own noses and to look outwards”.* This outward looking focus enabled them to articulate the broader implications for clinical innovation in supporting breastfeeding.

### Findings from the Project team

The following section describes the project team’s evaluation of the process under four themes. There was a realisation that the process was facilitated by good preparation and planning in order to build momentum.

**Building momentum**, that is, creating and sustaining forward movement, was evident throughout the project. At the beginning participants did not know where they were going, but once they were “activated” things became evident. “*When we meet, we can see*”. Initially there was a sense of “group think” with participants “not knowing why *they were there” and* facilitators trying to determine “*how best to engage/organise the group*”. In the early stages of the study the art and facilitated creativity enabled a feel-good factor that encouraged discussions and allowed *“thorny”* or *“prickly*” issues to come to the fore. However, it was a story of “*one step forward, two steps back”*, incremental positive steps were achieved but these were scattered with worries, occupying thoughts and disappointments along the way. The momentum swung from beginning at a “*distracted, harried place of being to a more open thoughtful and re-igniting way of thinking.”*

During the WBLGs and throughout the project, the team felt that they were **balancing** the groups from one WBLG to the next but also the diversity within the group. While the intention was to ensure there was a wide representation of all grades of practice and support staff, the reality was that there was *“disappointing engagement from some staff grades such as porters, catering*” predominantly due to scheduling conflicts e.g. WBLGs hosted at lunchtime. Facilitators therefore found it challenging to ensure such representation of all participants across the 9 WBLGs. However, an unexpected benefit of this was smaller groups which led to more critical discussion.

While participants spoke a lot about different environments they worked in, it was also evident that **space matters**, that is, the physical environment in which the WBLGs were hosted. Having a “*comfortable space*” was a key feature in the successful running of the WBLGs. Participants and facilitators reported that *“place matters”* and that when the space for WBLGs was uninviting it prevented “*good interaction*” and “*open discussion*”. Alternatively having a space where everyone could see one another and where it was possible to carry out the creative activities enabled better conversations and allowed *“ideas to flow*”.

**Being present** focused on attendance at the WBLGs and on the engagement, social atmosphere, and bonding of the groups both at the sessions and carrying out their workplace activities between sessions. A lot of *“energy”* was used by the facilitators and participants in “*lighting the fire”* and in maintaining the *“slow burn”* which led to practice changes. *“These were the outcomes of the fire”. “Energy”* was also expended by participants with “*trying to attend”* sessions, *“trying to engage*” other staff and families and “*trying to do better”* in practice. Facilitators expended energy in “*trying to increase attendance”* and in the case of the clinical research nurse, focused on staff recruitment and providing support to clinical sites throughout the study.

## Discussion

The findings presented in this paper are a core output of a larger project, the overarching aim of which is to enhance the implementation of evidence-based practice for Exclusive Breastfeeding throughout the pregnant woman’s journey to 3 months postpartum. While the overall project evaluation outcome data is reported elsewhere [54], the following discussion focuses on the findings as related to participants’ involvement and outcomes of that involvement in the project, in addition to the efficacy of using the PAR process and WBLGs for practice enhancement.

Clearly the reality of supporting exclusive breastfeeding within maternity, primary and community care settings is challenging. Like previous literature, empowerment was recognised as both an outcome in itself and as an intermediate step to being able to deliver improved health and well-being [55, 56]. It is also a process that helps people who are less powerful to identify problems, make decisions, and take action [57], in this case through teamwork, sharing experiences and facilitated envisioning of positive, impactful changes. This is also a crucial objective for women as they navigate their breastfeeding journey, while simultaneously transitioning to new motherhood [58]. Ensuring that it is the community, specifically mothers, and those supporting breastfeeding driving the process, is crucial for attaining sustainable outcomes. An example of this is the pioneering, visionary Community Mothers programme established in Ireland in 1983, developed as a support structure to enhance maternal and infant health [59]. Central to this home visiting community programme was peer support for breastfeeding (which was evaluated positively from parents’ perspective) as a sustainable model [56]. Key approaches within the project reflect the fact that successful breastfeeding interventions cannot simply be transferred or “standardised” across diverse populations but must be created within or adapted to local contexts by community members themselves [60].

Engaging the PAR process to prioritise participants’ views to identify and address priority issues led to incremental practice changes and the gathering of more detailed, nuanced data that may not have been acquired otherwise. These tangible outcomes were realised by building trust and connections across diverse settings, which helped to build or enhance existing constructive relationships. Furthermore, engaging and empowering stakeholders with differing attitudes, opinions, educational backgrounds and understandings of breastfeeding support contributed significantly to the findings. Similar to a PAR project reported by Tetui et al. [61], the presence of such heterogeny within groups significantly impacted the process. The involvement of stakeholders with varying levels of influence acted as a catalyst, triggering necessary changes within and across different health system contexts.

Given the complex organisational, social and political environments that PAR navigates within, the implementation of this process was neither smooth nor straightforward, aligning with experience previously reported [62, 63]. Consistent with literature pertaining to practice change and the PAR approach [38, 63–65] several challenges were identified in both participant involvement and facilitator support during the implementation evaluation. Participants struggled with balancing attendance at WBLGs and executing action plans amid existing work obligations. For example, catering staff struggled to attend the WBLGs which were scheduled during patient mealtimes. Yet they went beyond any requirement or expectation to attend scheduled training sessions during their annual leave. Facilitators and the broader research team often faced tensions between the timeline of the research project and its broader objectives. As supported by empirical research and discursive papers in this area, the PAR approach requires flexibility with timeframes, as achieving practice transformation largely depends on adapting to and aligning with participant progress [55, 64, 65].

To address the challenges in this project, strategies included expert facilitation, proficient management of WBLGs, ongoing team self-reflection, critique, and leveraging support by the clinical research nurse. The critical role of skilled facilitation in driving practice change is highlighted in the implementation evaluation meta-narrative. Harvey et al. [66] defined facilitation as aiding people in analysing current practices, leading to behavioural and work practice changes. Facilitation involved guided activities that encouraged creative thinking and challenged participants to reflect and problem-solve. This project employed distributed facilitation, with three facilitators overseeing each WBLG, each bringing expertise in subject content, health system context, and practice development skills. Recognising the need for facilitators to possess both topic and facilitation expertise [67], emphasis was also placed on context-sensitive facilitation across diverse health system settings [68]. This was implemented through the clinical research nurse who was integral in organising WBLGs and bridging local stakeholders and external facilitators. Such context-awareness was vital for engaging across various health sectors (community, primary, and acute care), enhancing understanding of different systems collaborating to support breastfeeding. Effective WBLG management involved thorough session preparation, including selecting suitable venues, advance scheduling, maintaining manageable group sizes, and precise record-keeping. Echoing Tetui et al. [61], smaller learning groups (about 8–10 participants) and a flexible learning space fostered interaction, openness, and concentration, leading to higher engagement levels.

### Strengths and limitations

This study is the first of its kind to explore the reality of supporting exclusive breastfeeding within maternity, primary and community care settings using a PAR approach. It provides new insights into factors which both hinder and enable the realisation of practical, ‘real-world,’ changes at the point of care, which can be used to inform developments needed within both at healthcare system and policy level for women and infants in Ireland and elsewhere. Notably, the type and extent of engagement a PAR approach requires ensures that any strategies developed are grounded in the realities of those who will be implementing and affected by these changes. To this end, PAR is a particularly suitable methodological lens through which to understand more effectively and address the challenges of translating breastfeeding policy to practice implementation.

Achieving data trustworthiness included democratic and sustained engagement with participants throughout the project duration and the use of data triangulation from several data sources (e.g., agendas, meeting minutes, facilitator debriefs and reflections). Reflexivity, in terms of examining one’s own conceptual lens, explicit and implicit assumptions, preconceptions and values, and how these affect research decisions, was ongoing throughout the study. These took the form of debriefs, developing agendas in response to the previous WBLG needs, and reflections that research team members wrote after conducting the WBLGs. The limitations of this study relate mostly to the fact that the recruitment of participants was confined to one geographical location with a specific healthcare provision model, thus limiting the application of findings beyond this context.

## Conclusions

This study emphasises the value of WBLGs and PAR to enhance the understanding and approach of diverse healthcare professionals and support workers, working in diverse settings, towards breastfeeding support in Ireland. Through innovative facilitation techniques and collaborative learning, it has identified preconceived ideas and practices in lactation support, encouraging participants to adopt new perspectives and strategies. The study has fostered a participatory environment where all ideas are valued equally, promoting ownership among participants, leading to a deeper mutual understanding of the challenges in breastfeeding support. These insights into continuous experiential learning among healthcare professionals are instrumental for potential improvements in the care of breastfeeding mothers and their babies. This research offers promising potential for future practice and policy development in maternal and child health, highlighting the impactful role of WBLGs and PAR in healthcare settings.

## Data Availability

The datasets used and/or analysed during the current study are available from the corresponding author on reasonable request.
